# After surviving cancer, what about late life effects of the cure?

**DOI:** 10.15252/emmm.201607062

**Published:** 2016-11-16

**Authors:** Julie Nonnekens, Jan HJ Hoeijmakers

**Affiliations:** ^1^ Department of Molecular GeneticsErasmus Medical CenterRotterdamThe Netherlands

**Keywords:** Cancer, Neuroscience

## Abstract

The widely used chemotherapeutic cisplatin causes ototoxicity as late‐term side effect. In this issue, Benkafadar *et al* (2017) decipher the mechanism of cisplatin‐induced ototoxicity and provide evidence that transient inhibition of p53 ameliorates ototoxicity without influencing chemotherapeutic efficacy. These findings may open exciting perspectives for reducing (late‐term) side effects of cancer treatment.

Cisplatin (CDDP) is a frequently employed chemotherapeutic drug both in curative and palliative settings. When cancer patients are cured due to CDDP therapy, they unfortunately often experience severe long‐term side effects including irreversible hearing loss (ototoxicity) and permanent neuronal and renal damage. CDDP targets the DNA by bivalently reacting with purine bases mainly G residues, generating inter‐ and intra‐strand crosslinks (Fig 1) (Fram, 1992). These adducts disrupt DNA helical structure, activate the DNA damage response (DDR), and interfere with vital DNA transactions, obstructing transcription and replication. Particularly inter‐strand cross‐links, preventing strand separation, are highly toxic in proliferating cells, which includes tumor cells but also normal tissues in the body. Handling of these lesions by cellular repair systems or upon blocking transcription and replication may result in the formation of DNA double‐strand breaks and other detrimental DNA intermediates. In replicating cells (inadequate) repair of CDDP lesions may lead to mutations (base substitutions and chromosomal aberrations), which hamper cell function and contribute to secondary tumors later in life. In non‐proliferating cells, it is likely that persisting inter‐ and intra‐strand cross‐links or irreparable double‐strand breaks cause chronic activation of the DDR and enduring, local suppression of gene expression, affecting cell function. The DDR is triggered by several signaling proteins such as Ataxia telangiectasia mutated (ATM) and AT‐ and rad3‐related (ATR) that are recruited to the site of DNA damage where they initiate a cascade of local chromatin modifications acting as a platform for binding of numerous repair proteins to facilitate repair. Simultaneously, downstream proteins promote transient cell cycle arrest and, in case of failing repair, cell death via apoptosis or necroptosis, or cellular senescence which may lead to cellular dysfunction and genome instability. The tumor suppressor protein p53 is a key player in these decisions, responsible for most of these outcomes, which influence long‐term effects (Fig 1) (Jackson & Bartek, 2009).

**Figure 1:**
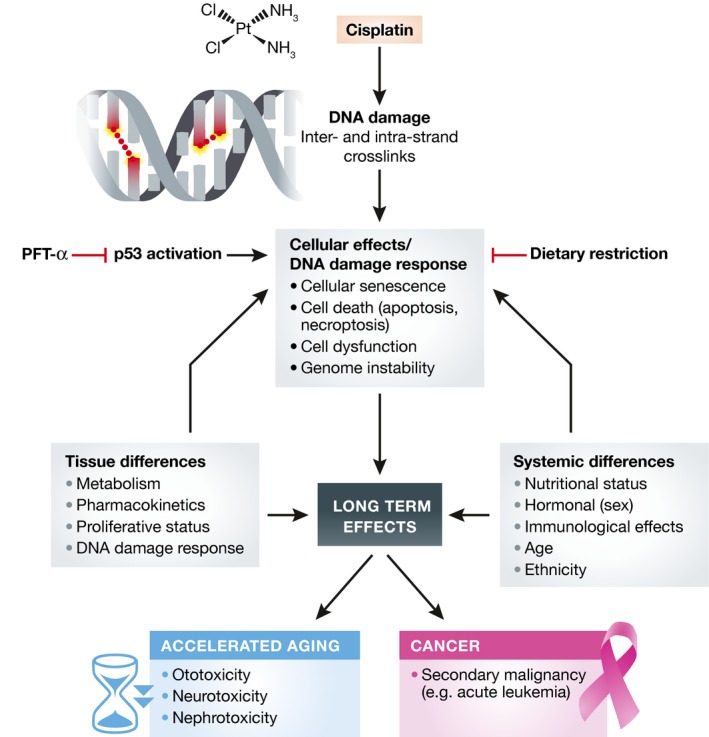
Factors influencing short‐ and long‐term effects of cisplatin Overview of how different factors can influence long‐term normal tissue effects after cisplatin (CDDP) treatment. CDDP causes DNA damage. Many cellular effects are mediated by p53 as part of the DNA damage response yielding long‐term side effects. Factors such as tissue differences (metabolism, pharmacokinetics, proliferative status, and DNA damage response) and systemic differences (nutritional status, hormones, immunological effects, age, ethnicity, etc.) influence these side effects, which resemble accelerated aging (ototoxicity, neurotoxicity, and nephrotoxicity) and/or increase cancer (secondary malignancies). p53 inhibition using PFT‐α and likely dietary restriction might reduce at least part of these side effects.

These late life events consequent to anticancer treatment include cardiac, hearing, vision, respiratory, and renal problems, all resembling aspects of aging in a premature manner (Armstrong *et al*, 2014). In addition, cancer survivors display other signs of accelerated aging, such as fatigue and physical and memory impairment (Maccormick, 2006). This is in line with increasing evidence for a strong link between accumulating DNA damage and acceleration of aging (Hoeijmakers, 2009). For instance, chronic exposure of mice to a low dose of gamma irradiation accelerates aging. Also most human progeroid syndromes are due to mutations in genome stability genes, specifically DNA damage repair pathways. Similarly, mouse models with defects in DNA repair and response systems that primarily protect from the cytotoxic or cytostatic effects of DNA damage display many *bona fide* features of accelerated aging in multiple organs and tissues at histopathological, physiological, hormonal, and functional levels (Hoeijmakers, 2009).

Ototoxicity by CDDP is caused by degeneration of neuro‐sensory epithelium of the cochlea mostly by damage to the outer hair cells (OHCs). Until now, no molecular pathway underlying the CDDP‐induced ototoxicity has been described. In this issue, Benkafadar *et al* (2017) show that the ATM‐Chk2‐p53 axis of the DDR is highly activated in OHCs after CDDP exposure. Interestingly, targeting this pathway in mice using a genetic approach or a pharmacological inhibitor of p53 (Pifithrin‐α or PFT‐α) preserves hearing function by preventing cochlear cell death (Benkafadar *et al*, 2017), showing that influencing the DDR can diminish long‐term consequences and keeping OHCs alive after CDDP treatment may reduce hearing loss. Importantly, the authors also show that inhibition of p53 using PFT‐α did not go at the expense of chemotherapeutic efficacy. These findings can have important implications for reducing side effects of cisplatin and likely other cancer therapies based on injuring DNA. Late life damage to other tissues is most likely also mediated via the DDR including p53. Application of PFT‐α might therefore not only improve hearing abilities, but also diminish other side effects as nephrotoxicity and neurotoxicity. However, genome instability caused by chemotherapy may also cause secondary malignancies (Fig 1). Transient inhibition of the p53 pathway might increase this risk, and therefore, it is critical to study this in detail. Another aspect related to DNA damage and p53 activity is accumulation of senescent cells in many tissues due to chemotherapy. These persistent cells, carrying irreparable DNA damage, exhibit a so‐called senescence‐associated secretory phenotype (SASP), excreting numerous factors, including a litany of pro‐inflammatory cytokines, such as IL‐6, IL‐8, IL‐1α, TNFβ, and others (Childs *et al*, 2015), which likely has systemic effects and may contribute to, for example, fatigue often observed after chemotherapy. Recently, selective elimination of senescent cells, which also accumulate in normal aging, has been reported to improve several health parameters in mice (Childs *et al*, 2015). It will be important to determine what happens to senescence and to fatigue after transient p53 inhibition.

In a wider context, tissue differences in metabolism (including enzymatic processing of the agent), pharmacokinetics, proliferative versus post‐mitotic status, perfusion, blood–brain barrier, and efficacy of the DDR contribute to heterogeneity in response to chemotherapy next to genetic (e.g., ethnic) differences. Several of these parameters are also influenced by hormonal (e.g., gender) and immune status, age and even circadian time. For instance, upon aging, the p53 response gets down‐regulated in many tissues (Feng *et al*, 2007), influencing response to chemo‐ and radiotherapy. An important not yet generally appreciated factor, with significant therapeutic implications is nutrition. Various recent studies have shown that in particular dietary restriction (DR, i.e., reduction in food intake without malnutrition) or short‐term fasting may have remarkable clinical potential in reducing short‐ and perhaps long‐term side effects. DR is for long known to extend life span in numerous species by redirecting energy resources from growth to maintenance and defense mechanisms, including stress resistance and antioxidant systems. DR has been shown to reduce damage due to ischemia–reperfusion, and currently, clinical trials are ongoing to examine whether it also reduces short‐ and long‐term side effects of chemotherapy without compromising anticancer efficacy (Brandhorst & Longo, 2016). Also inhibitors of necroptosis may have a potential to reduce short‐ and long‐term side effects (Matt & Hofmann, 2016). It might be interesting to examine these interventions in combination with transient inhibition of p53.

In summary, CDDP treatment causes many severe (late stage) side effects that are tissue specific and resemble accelerated aging. Pharmacological inhibition of p53 and other interventions might significantly reduce side effects, thereby significantly improving quality of life of cancer patients.
